# Aneuploidy Mediates Rapid Adaptation to a Subinhibitory Amount of Fluconazole in Candida albicans

**DOI:** 10.1128/spectrum.03016-22

**Published:** 2023-02-28

**Authors:** Liu-liu Sun, Hao Li, Tian-hua Yan, Ting Fang, Hao Wu, Yong-bing Cao, Hui Lu, Yuan-ying Jiang, Feng Yang

**Affiliations:** a Department of Pharmacology, Shanghai Tenth People’s Hospital, Tongji University School of Medicine, Shanghai, China; b Department of Physiology and Pharmacology, School of Basic Medicine and Clinical Pharmacy, China Pharmaceutical University, Nanjing, China; c Institute of Vascular Disease, Shanghai TCM-Integrated Hospital, Shanghai University of Traditional Chinese Medicine, Shanghai, China; The Ohio State University

**Keywords:** aneuploidy, antifungal resistance, antifungal tolerance, *Candida albicans*, fitness, fluconazole

## Abstract

Candida albicans is a prevalent, opportunistic, human fungal pathogen. Antifungal drug resistance and tolerance are two distinct mechanisms of adaptation to drugs. Studies of mechanisms of drug resistance are limited to the applications of high doses of drugs. Few studies have investigated the effects of subinhibitory amounts of drugs on the development of drug resistance or tolerance. In this study, we found that growth in a subinhibitory amount of fluconazole (FLC), a widely used antifungal drug, for just a short time was sufficient to induce aneuploidy in C. albicans. Surprisingly, the aneuploids displayed fitness loss in the presence of subinhibitory FLC, but a subpopulation of cells could tolerate up to 128 μg/mL FLC. Particular aneuploidy (ChrR trisomy) caused tolerance, not resistance, to FLC. In the absence of FLC, the aneuploids were unstable. Depending on the karyotype, aneuploids might become completely euploid or maintain particular aneuploidy, and, accordingly, the tolerance would be lost or maintained. Mechanistically, subinhibitory FLC was sufficient to induce the expression of several *ERG* genes and as well as the drug efflux gene *MDR1*. Aneuploids had a constitutive high-level expression of genes on and outside the aneuploid chromosomes, including most of the *ERG* genes as well as the drug efflux genes *MDR1* and *CDR2*. Therefore, aneuploids were prepared for FLC challenges. In summary, aneuploidy provides a rapid and reversible strategy of adaptation when C. albicans is challenged with subinhibitory concentrations of FLC.

**IMPORTANCE** Genome instability is a hallmark of C. albicans. Aneuploidy usually causes fitness loss in the absence of stress but confers better fitness under particular stress conditions. Therefore, aneuploidy is considered to be a double-edged sword. Here, we extend the understanding of aneuploidy. We found that aneuploidy arose under weak stress conditions but that it did not confer better fitness to the stress. Instead, it was less fit than its euploid counterparts. If the stress was withdrawn, aneuploidy spontaneously reverted to euploidy. If the stress became stronger, aneuploidy enabled subpopulation growth in a dose-independent manner of the stress. Therefore, we posit that aneuploidy enables the rapid and reversible development of drug tolerance in C. albicans. Further studies are required to investigate whether this is a general mechanism in human fungal pathogens.

## INTRODUCTION

*Candida* species are the most common human fungal pathogens and are the fourth leading cause of nosocomial bloodstream infections in the United States (1). Among them, C. albicans is the most common fungal pathogen that is responsible for nosocomial systemic infections and is the most commonly isolated pathogen from clinical samples obtained from mucous membranes (2). C. albicans is a member of the human microbiota that inhabits the mouths, vaginal tracts, and gastrointestinal tracts of most healthy adults as a harmless commensal. However, it also causes diseases that range from superficial mucosal infections to life-threatening systemic infections (3).

Fluconazole (FLC) is a widely used antifungal drug that selectively inhibits the activity of cytochrome P450-dependent C-14 lanosterol demethylase, which is a key enzyme in ergosterol biosynthesis in fungi (4). FLC results in ergosterol depletion, membrane disruption, increased permeability, the leakage of cytoplasmic contents, and, ultimately, cell death (5). Simultaneously, FLC causes the accumulation of toxic 14-methyl sterols, especially 14α-methyl-3,6-diol (6). The most frequently observed mechanisms of resistance to FLC involve the following. (i) An increase in the amount of drug target. The C. albicans target of FLC is encoded by *ERG11*. The exposure of C. albicans to FLC induces the expression of *ERG* genes, including *ERG11* (7). The overexpression of *ERG11*, as well as other *ERG* genes, is associated with FLC resistance (8). (ii) An increased drug efflux (e.g., Cdr1p, Cdr2p, and Mdr1p). Cdr1p and Cdr2p belong to the ATP-binding cassette (ABC) superfamily, and Mdr1p belongs to the major facilitator superfamily (MFS). The overexpression of *CDR1*, *CDR2*, and *MDR1* are associated with FLC resistance (9, 10). (iii) An alteration to the drug binding site (8).

The effects of inhibitory amounts of FLC, as well as other antifungal drugs, in selecting for drug-resistant mutants have been well-studied (reviewed in references [8] and [11]). However, the concentrations of drugs that reach infecting pathogens can be subinhibitory but may nonetheless promote the emergence of drug resistance. In bacteria, even extremely low concentrations of antibiotics can select for resistant bacteria (12–14).

A further mechanism of adaptation to FLC, namely, drug tolerance, was recently identified in C. albicans. The Berman lab found that a subpopulation of C. albicans cells could overcome drug stress and grow slowly at supra-MICs of FLC (15). Unlike resistance, which is usually due to genetic mutations, tolerance is due to physiological or epigenetic mechanisms (16). Previously, we also found that tolerance to ketoconazole, another azole, was due to physiological factors, including the medium and the temperature, in C. albicans (17).

Resistance and tolerance can be measured using disk diffusion assays (DDAs). In DDAs, a customed script, namely, diskImageR, was developed by the Berman lab to analyze the photographs of the plates (18). The degree of drug resistance is determined by the radius of inhibition (RAD), and tolerance is determined by the fraction of growth (FoG) within the zone of inhibition. Usually, 20% drug inhibition (RAD_20_ and FoG_20_) is used to measure resistance and tolerance, respectively (15, 18).

In this study, we investigated the effect of short-time exposure to subinhibitory concentrations of FLC on the development of drug resistance and tolerance in C. albicans. We found a novel genetic mechanism of FLC tolerance via unstable aneuploids. Combined with the DNA-seq and RNA-seq of the aneuploids, we studied the molecular mechanism of drug adaptation and the resulting gain of drug tolerance.

## RESULTS

### Short-time exposure to subinhibitory concentrations of fluconazole selects progeny which tolerate inhibitory concentrations of fluconazole.

**FIG 1:**
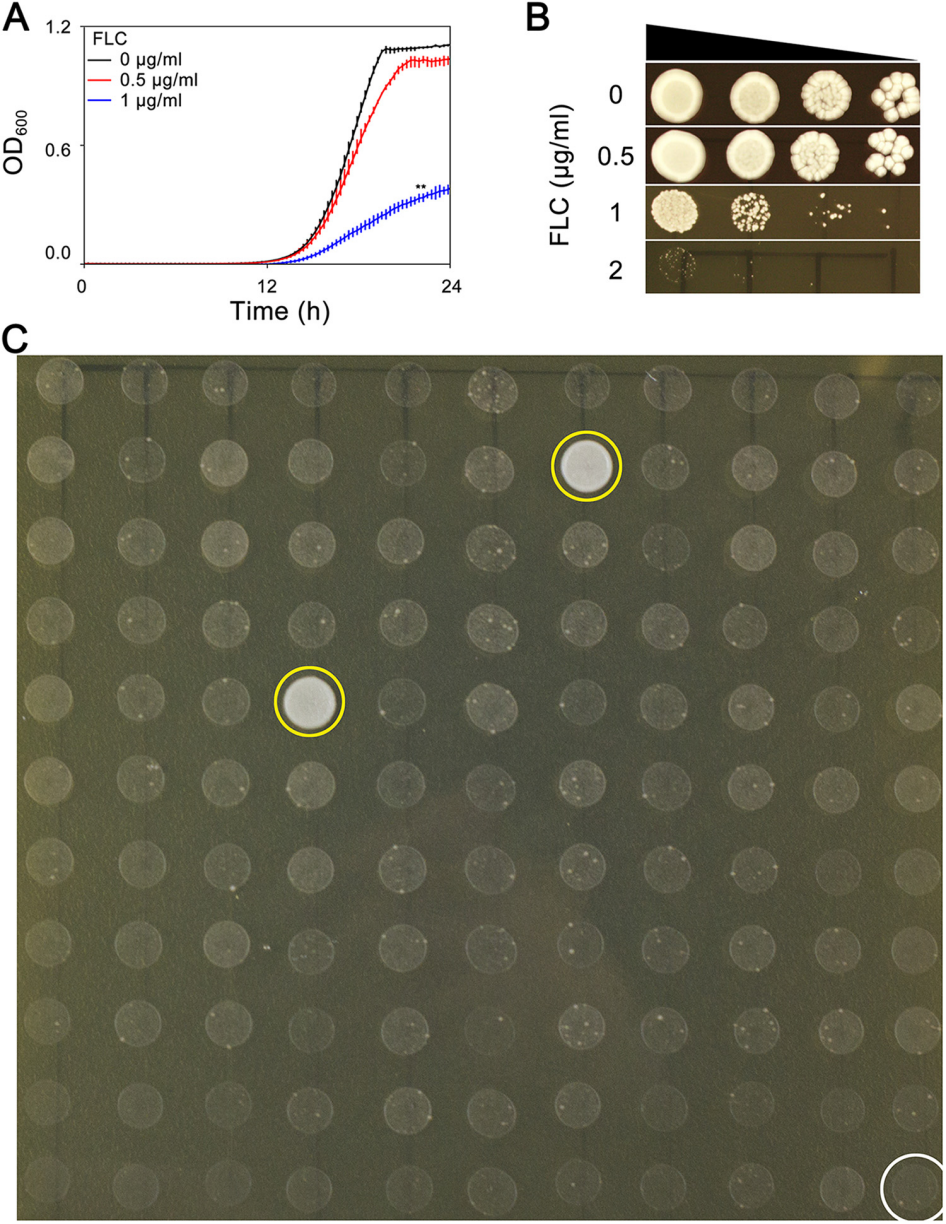
Obtaining fluconazole adaptors. Susceptibility of the lab strain SC5314 to fluconazole (FLC) was tested with a growth curve (A) and a spot assay (B). In panel A, approximately 2.5 × 10^3^ cells/mL of SC5314 in 150 mL YPD with or without FLC were incubated in a 96-well plate at 30°C. The optical density at 600 nm (OD_600_) was monitored by a plate reader (Infinite F200 PRO; Tecan, Switzerland) at 15 min time intervals for 24 h. The data are presented as the mean ± SD of three biological replicates. In panel B, the cell densities were adjusted to 1 × 10^7^ cells/mL, and 3 μL of 10-fold serial dilutions were spotted on YPD agar plates supplemented with FLC. The plates were incubated at 30°C for 48 h and then photographed. In panel C, SC5314 was grown in YPD + 0.5μg/ml FLC. After 24h, randomly 120 colonies were tested with spot assay for tolerance to FLC. White circle indicates the parent. Yellow circles indicate two tolerant adaptors.

The ability of lab strain SC5314 to grow in the presence of FLC was investigated in both YPD broth and on YPD agar. In YPD broth, growth was not significantly inhibited at 0.5 μg/mL FLC (Fig. 1A). On YPD agar plates, growth was obviously inhibited at 2 μg/mL FLC (Fig. 1B). We investigated whether exposure to 0.5 μg/mL FLC for a short time was sufficient to observe tolerance and further facilitate growth at an inhibitory concentration of FLC, namely, 2 μg/mL. SC5314 was grown in the presence of 0.5 μg/mL FLC for 24 h. Randomly, 120 colonies were tested. 2 of these colonies (FY1284 and FY1285) could then grow at 2 μg/mL FLC (Fig. 1C, yellow circles), whereas the rest of the 118 colonies, as well as the parent, could not (Fig. 1C, white circles). As a control, none of the 120 colonies that were pregrown in YPD broth for 24 h were tolerant to FLC (Fig. S1). Therefore, short term exposure to a subinhibitory concentration of FLC enabled the appearance of rare adaptors which could tolerate an inhibitory concentration of FLC.

### The adaptors have fitness loss at subinhibitory concentrations of fluconazole.

**FIG 2:**
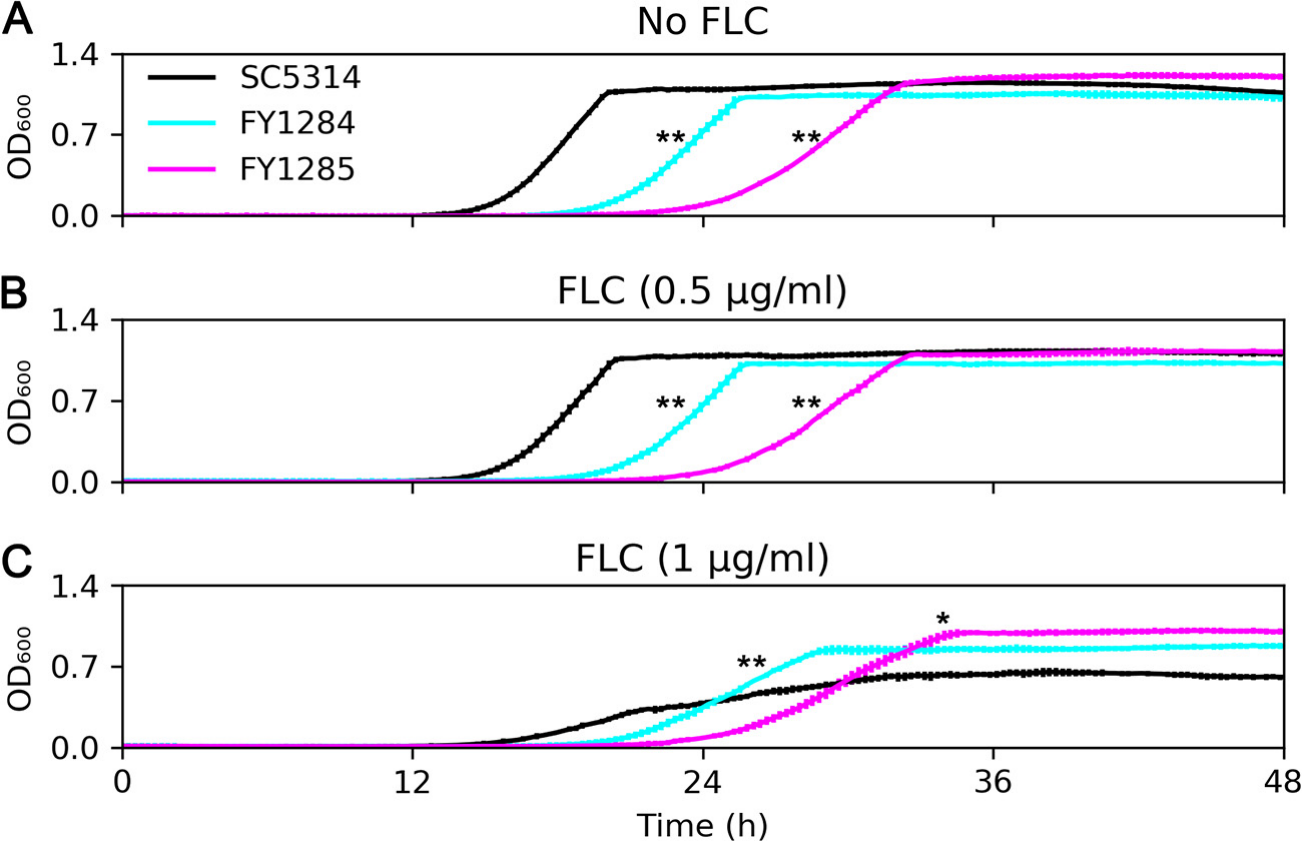
Fitness assay of fluconazole adaptors. The fitness of the FLC adaptors FY1284 and FY1285 was compared to that of parent SC5314 by measuring the growth curves in YPD broth (A) or in YPD broth supplemented with subinhibitory (B), or inhibitory (C) FLC. The data are presented as the mean ± SD of three biological replicates. * and ** indicate *P* values of *P* < 0.05, and *P* < 0.001, respectively, as determined by two-tailed tests of the slopes of the curves.

Next, we investigated whether the two adaptors obtained tolerance to FLC at a fitness cost in the absence of FLC. Both adaptors grew significantly slower than did the parent in YPD (Fig. 2A). Surprisingly, both adaptors were also significantly less fit than the parent in YPD with 0.5 μg/mL FLC (Fig. 2B); however, they were significantly more fit than the parent in YPD supplemented with 1 μg/mL FLC (Fig. 2C). Therefore, although the two adaptors were selected at subinhibitory concentrations of FLC and had better fitness in the presence of an inhibitory concentration of FLC, they had fitness loss in the absence of FLC or at subinhibitory concentrations of FLC.

### Tolerance to a wide range of concentrations of fluconazole is due to subpopulation growth.

**FIG 3:**
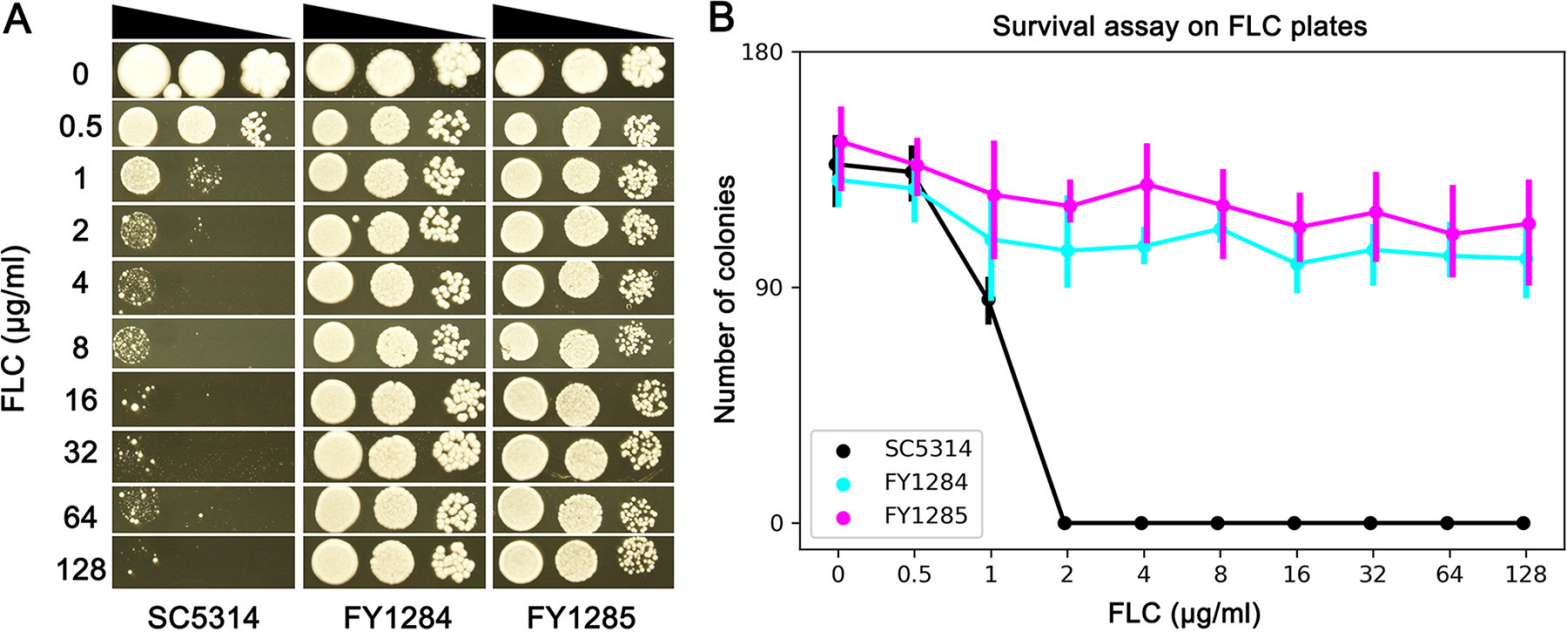
A subpopulation of fluconazole adaptors displays a dose-independent tolerance to fluconazole. The extent of tolerance to FLC was measured by spot assay (A) and a survival assay (B), using a wide range of FLC concentrations (0.5 to 128 μg/mL, 2-fold increase). In panel A, the plates were incubated at 30°C for 3 days and then photographed. In panel B, approximately 200 cells of each test strain were spread on YPD plates supplemented with or without FLC. After 3 days of incubation at 30°C, the number of colonies on each plate were counted. The data are represented as the mean ± SD of three technical replicates.

We used two assays to investigate the extent to which tolerance enabled growth in the presence of FLC. In a spot assay, while the parent could only grow at FLC concentrations of less than 2 μg/mL, both adaptors could grow at supra-MIC FLC concentrations that ranged from 2 μg/mL to 128 μg/mL FLC (Fig. 3A). In a survival assay, we calculated the percentage of colonies on drug plates, compared to those on plates without a drug. The parent showed FLC dose-dependent growth. Approximately 62.2% of the parental cells that were spread on a YPD plate with 1 μg/mL grew, compared to those observed when grown on YPD without FLC. On plates with larger amounts of FLC, no colonies appeared (Fig. 3B). FY1284 and FY1285 showed FLC dose-independent growth. On YPD plates with 1 μg/mL to 128 μg/mL FLC, FY1284 displayed survival rates between 75.6% and 85.8%, and FY1285 displayed survival rates between 76.1% and 89.2% (Fig. 3B). Therefore, tolerance enabled dose-independent sub-population growth at supra-MICs of FLC.

### The adaptors do not have an elevated fluconazole MIC.

**FIG 4:**
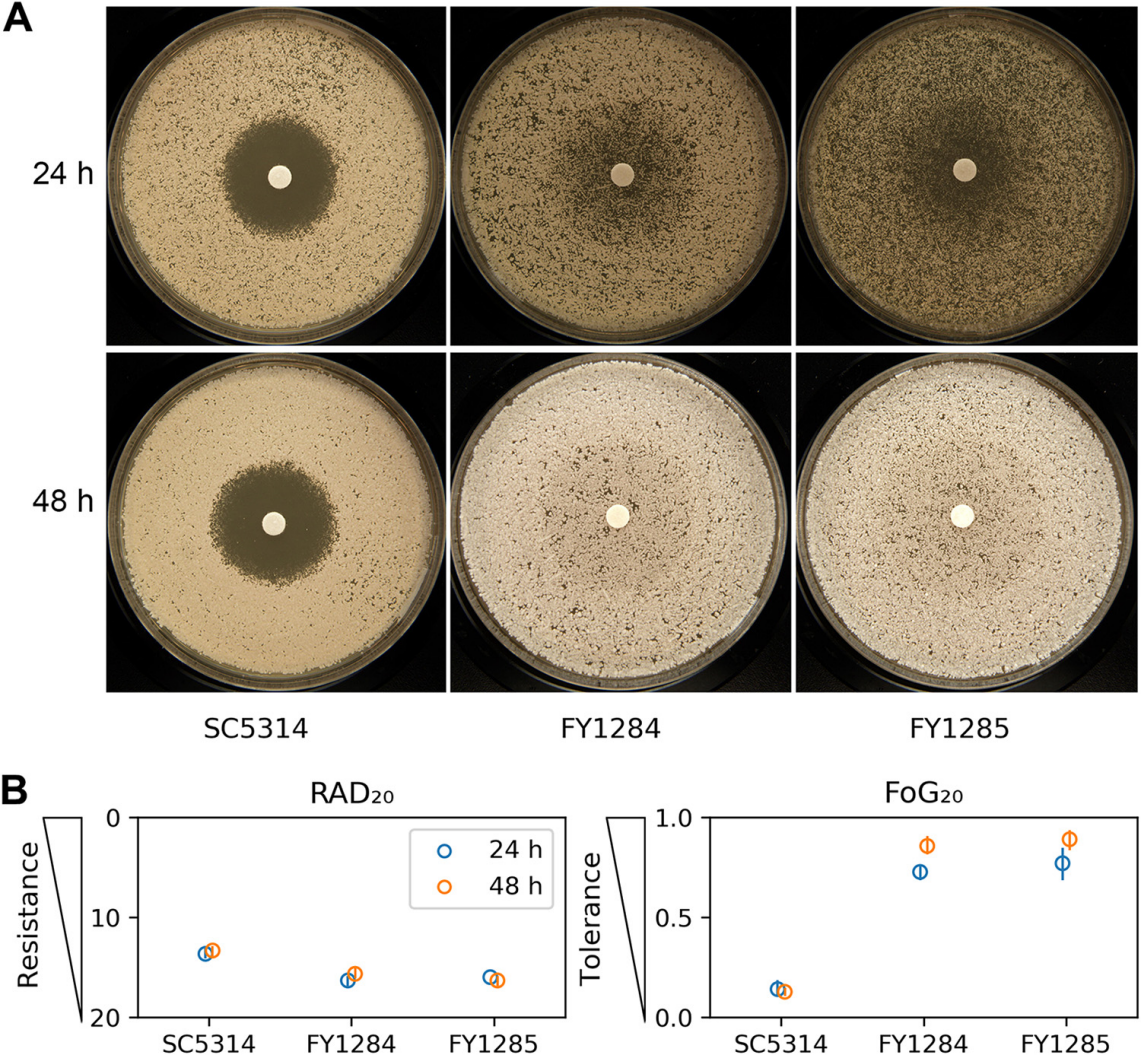
Evaluation of fluconazole resistance and tolerance via a disk diffusion assay. In a disk diffusion assay (A), for each test strain, the same number of cells (approximately 1 × 10^5^ cells) were spread on YPD plates. The disks contained 25 μg FLC. The plates were incubated at 30°C for 24 h and 48 h and then photographed, respectively. The pictures shown in panel A are 24 h and 48 h growth plates. In panel B, the photographs were analyzed using diskImageR. The RAD_20_ and FoG_20_ values were measured at both 24 h and 48 h. The RAD_20_ and FoG_20_ values are presented as point plots that were generated using a custom Python script. The circles represent the means, and the vertical lines inside the circles represent the standard deviations from three biological repeats.

We investigated whether the adaptors acquired resistance or tolerance to FLC. DDAs were performed (Fig. 4A), and *diskImageR* was used to analyze the data (Fig. 4B). *diskImageR* calculates RAD_20_ and FoG_20_ as measurements of resistance and tolerance, respectively (15, 18). At 24 h, the RAD_20_ values of the adaptors FY1284 and FY1285 were 16.33 ± 0.58 and 16.00 ± 0.00, respectively, whereas the RAD_20_ value of the parent was 13.67 ± 0.58. The FoG_20_ values of FY1284 and FY1285 were 0.73 ± 0.03 and 0.77 ± 0.08, respectively, and the FoG_20_ value of the parent was 0.14 ± 0.04. At 48 h, the RAD_20_ values of FY1284, FY1285, and SC5314 were 15.67 ± 0.58, 16.33 ± 0.58, and 13.33 ± 0.58, respectively, and the FoG_20_ values of FY1284, FY1285, and SC5314 were 0.86 ± 0.04, 0.89 ± 0.05, and 0.13 ± 0.02, respectively. Since the RAD_20_ is inversely proportional to the MIC, surprisingly, the parent was therefore more resistant to FLC than were the two adaptors. However, the adaptors obviously obtained tolerance to FLC. Note that in this study we used YPD-agar medium, on which the parent SC5314 was not tolerant (19). However, on Casitone-agar medium, SC5314 was tolerant (15).

### Tolerance is due to aneuploidy.

**FIG 5:**
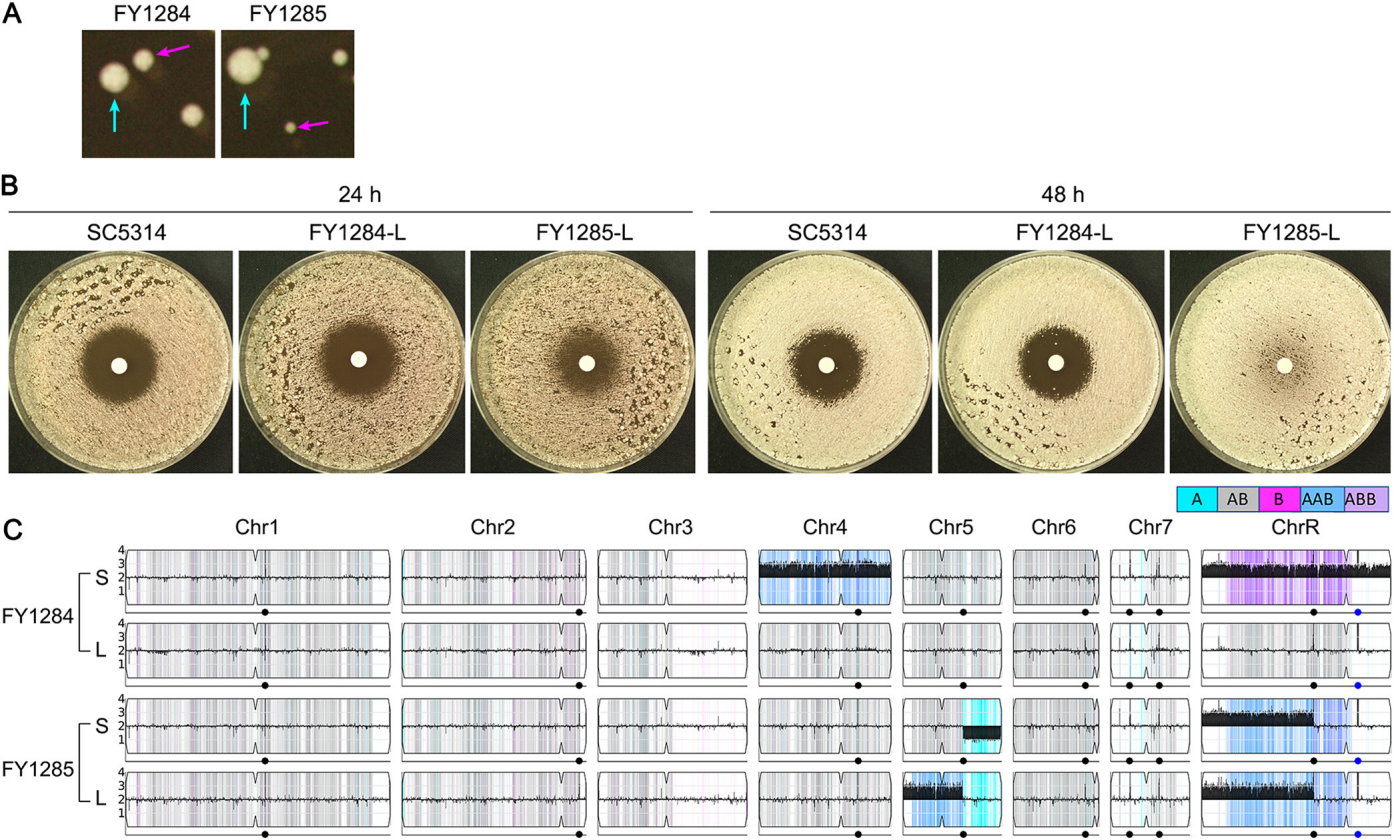
Instability of fluconazole adaptors. Colony instability was evaluated by observing colony size variations on YPD plates (A). Magenta arrows indicate small (S) colonies. Cyan arrows indicate large (L) colonies. Phenotypic instability was evaluated by performing disk diffusion assays of the large colonies (FY1284-L and FY1285-L) for their tolerance to FLC (B). The disks contained 25 μg FLC. Genomic instability was evaluated via the whole-genome sequencing of both the S and L colonies. The sequencing data were visualized using Ymap (C). The read depth (normalized to that of the diploid parent) is shown on the *y* axis on a log_2_ scale, converted to absolute copy numbers (1–4). Allelic ratios (A:B) are color-coded: gray, 1:1 (A/B); cyan, 1:0 (A or A/A); magenta, 0:1 (B or B/B); purple, 1:2 (A/B/B); blue, 2:1 (A/A/B).

Both adaptors were unstable on YPD plates yielding small (magenta arrow) and large (cyan arrow) colonies (Fig. 5A). One small and one large colony were randomly chosen and plated on YPD plates, respectively. The small colonies (FY1284-S and FY1285-S, respectively) and the large colonies (FY1284-L and FY1285-L, respectively) were collected and sequenced.

FY1284-S, the small colony of adaptor FY1284, had duplications of whole Chr4 (AAB) and ChrR (ABB). FY1284-L, the large colony of adaptor FY1284, lost extra copies of Chr4 and ChrR. FY1285-S was also aneuploid. It had segmental loss (approximately 0.46 Mb) on the right arm of Chr5 and segmental duplication (approximately 1.35 Mb) on the left arm of ChrR. However, FY1285-L maintained segChrRx3 and lost SegChr5x1, but it gained SegChr5x3 (approximately 0.72 Mb) on the left arm (Fig. 5B). Therefore, the adaptor with whole chromosome aneuploidy was unstable in the absence of stress and reverted to euploidy by losing extra chromosomes. The aneuploid adaptor with segmental aneuploidy was also unstable, but, depending on the chromosome, it could maintain the aneuploid chromosome or gain compensatory duplication of another region on the aneuploidy chromosome.

We investigated whether the tolerance to FLC was also unstable. Measured at 24 h, FY1284-L had RAD_20_ and FoG_20_ values of 14.50 ± 0.71 and 0.14 ± 0.05, respectively, and FY1285-L had RAD_20_ and FoG_20_ values of 11.5 ± 0.71 and 0.39 ± 0.12, respectively, whereas the parent SC5314 had RAD_20_ and FoG_20_ values of 14.00 ± 1.41 and 0.13 ± 0.04, respectively. At 48 h, FY1284-L had RAD_20_ and FoG_20_ values of 14.00 ± 0.58 and 0.17 ± 0.02, respectively, and FY1285-L had RAD_20_ and FoG_20_ values of 10.33 ± 0.58 and 0.84 ± 0.18, respectively, whereas the parent SC5314 had RAD_20_ and FoG_20_ values of 13.33 ± 0.58 and 0.15 ± 0.01, respectively. Therefore, FY1284-L lost tolerance, and FY1285-L maintained tolerance (Fig. 5C). Furthermore, FY1285-L also became more resistant to FLC. This is consistent with the finding that the trisomy of left arm of Chr5 confers FLC resistance (20, 21). Since FY1284-S was tolerant and had Chr4x3+ChrRx3, whereas FY1284-L was not tolerant and was euploid, we concluded that the duplication of Chr4 and ChrR caused tolerance to FLC in FY1284. Both FY1285-S and FY1285-L had SegChrRx3. So, we concluded that ChrRx3 conferred tolerance to FLC in FY1285 and that the segmental trisomy of Chr5 conferred resistance to FLC.

### Aneuploid adaptors display the constitutive induction of *ERG* genes and efflux genes.

As shown in Fig. 1, the FLC MIC of SC5314 was 1 μg/mL. When cells were grown in YPD broth supplemented with a subinhibitory concentration of FLC (0.5 μg/mL), there was no significant change in growth, compared to growth in YPD broth. It was intriguing why some cells mutated to become aneuploid. Therefore, we compared the transcriptome of cells grown in YPD broth supplemented with 0.5 μg/mL FLC to cells grown in the absence of the drug. There were 284 significantly differential genes (*q* < 0.05): 172 genes were upregulated, and 112 genes were downregulated (Table S1). A GO analysis indicated that genes involved in ergosterol biosynthesis were significantly enriched in the upregulated genes, including *ERG11*, which encodes the target protein of FLC (Table 1; Table S2). In addition, among the genes associated with drug efflux, *MDR1*, which encodes a multidrug resistance protein of the major facilitator superfamily, was induced by FLC. However, CDR1 and CDR2, which encode the multidrug transporter of the ATP-binding cassette (ABC) superfamily, were not induced. Therefore, subinhibitory FLC was sufficient to induce the expression of the *ERG* genes and the drug efflux gene *MDR1*.

**TABLE 1:** Expression of genes involved in ergosterol biosynthesis and efflux

Gene	Chromosome	Ratio (allele A; allele B)		
		Sub-MIC treatment/no treatment	FY1284/SC5314	FY1285/SC5314
Genes involved in ergosterol biosynthesis				
*ERG1*	Chr1	1.95; 1.91	1.82; 1.76	1.13; 1.02
*ERG2*	Chr1	1.26; 1.33	2.29; 2.30	1.70; 1.74
*ERG3*	Chr1	1.41; 1.68	1.08; 1.30	0.62; 0.59
*ERG12*	Chr1	0.97; 1.00	1.16; 1.00	1.07; 1.01
*HMG1*	Chr1	1.00; 0.97	1.04; 0.73	1.17; 0.91
*MVD1*	Chr1	0.88; 0.88	0.94; 0.94	1.10; 1.10
*UPC2*	Chr1	1.16; 1.30	1.25; 1.87	0.78; 1.23
*ERG7*	Chr2	1.41; 1.56	0.91; 1.10	0.91; 1.06
*ERG9*	Chr2	1.39; 1.56	1.86; 2.07	1.57; 1.56
*ERG10*	Chr2	1.61; 1.60	2.02; 2.16	1.88; 2.03
*ERG20*	Chr2	0.95; 1.04	1.33; 1.04	1.61; 1.33
*ERG24*	Chr2	1.77; 1.67	1.38; 1.23	1.35; 1.16
*ERG4*	Chr3	1.26; 1.26	0.73; 0.70	0.73; 0.61
*ERG6*	Chr3	2.02; 2.07	3.25; 3.22	1.79; 1.65
*ERG8*	Chr4	1.03; 0.98	2.69; 1.36	1.81; 1.63
*ERG26*	Chr4	1.59; 1.59	2.74; 2.74	1.86; 1.86
*IDI1*	Chr4	1.09; 1.00	4.52; 2.08	1.91; 1.78
*ERG11*	Chr5	2.00; 1.97	3.52; 3.75	2.01; 2.12
*ERG5*	Chr7	1.50; 1.50	1.32; 1.32	1.06; 1.06
*ERG13*	ChrR	1.23; 1.24	1.24; 1.24	1.20; 1.20
*ERG25*	ChrR	1.59; 1.24	1.38; 2.96	1.63; 0.91
*ERG27*	ChrR	1.55; 1.55	3.24; 3.24	2.87; 2.87
Genes involved in efflux				
*CDR1*	Chr3	0.87; 0.69	0.81; 0.80	0.56; 0.57
*CDR2*	Chr3	1.11; 1.11	3.26; 3.26	3.06; 3.06
*MDR1*	Chr6	1.44; 1.43	1.22; 1.55	3.18; 4.14
*TAC1*	Chr5	1.17; 0.94	0.88; 0.97	0.83; 0.74
*MRR1*	Chr3	1.00; 1.00	0.74; 0.74	1.10; 1.10

**FIG 6:**
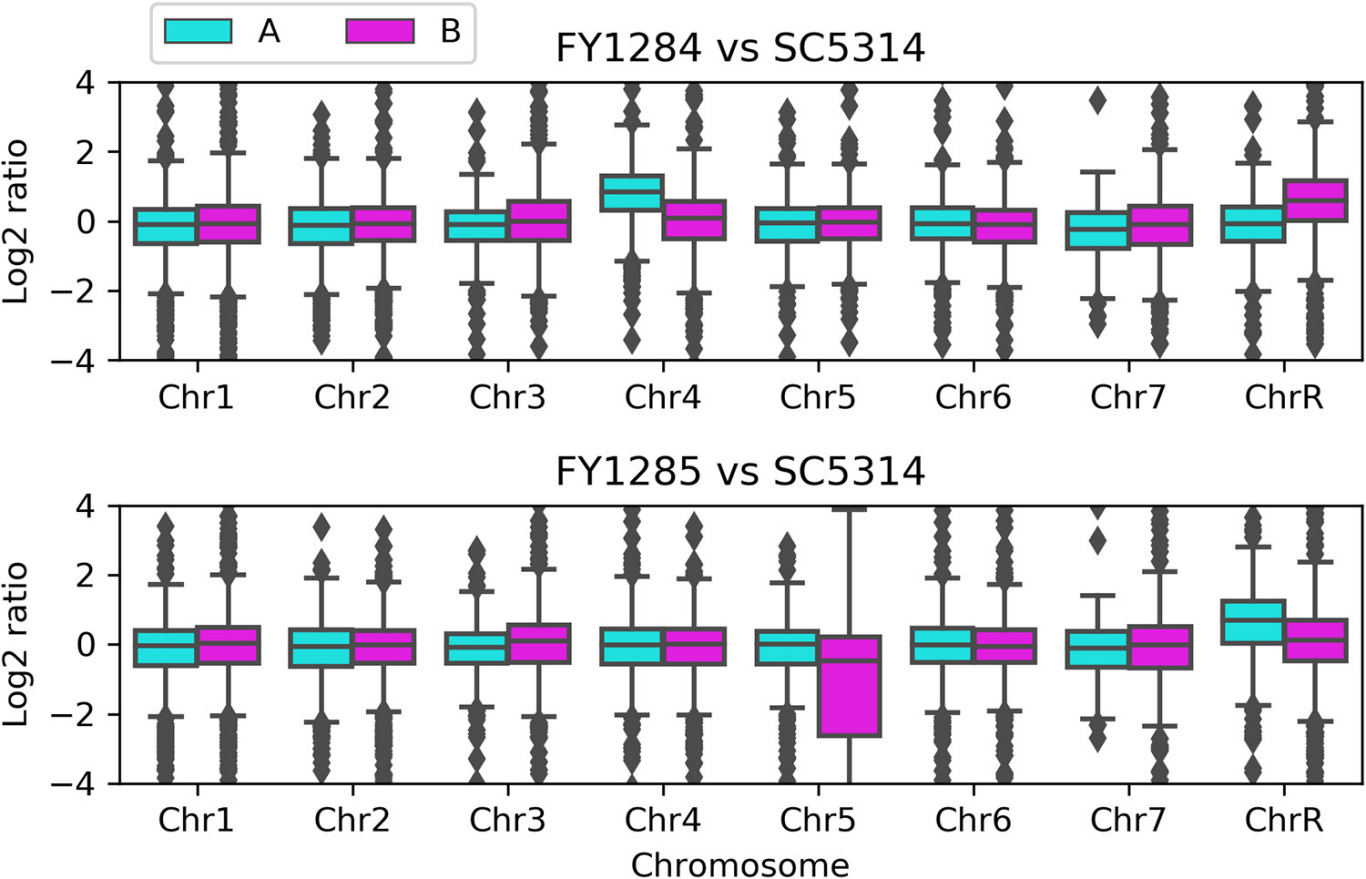
Aneuploidy caused the proportional elevated expression of genes on the aneuploid chromosomes. The transcriptomes of the FLC adaptors FY1284 and FY1285 were compared to the parent SC5314. The log_2_ ratios were plotted as a function of the chromosome and homolog (homolog A and homolog B) positions.

Next, we compared the transcriptome of the aneuploid adaptors to the wild-type strain. In general, genes on the trisomic or monosomic chromosomes had proportionally higher or lower expression than did genes on diploid chromosomes. Furthermore, on the trisomic chromosomes, genes on the duplicated homolog had an overall elevated expression (Fig. 6). Therefore, aneuploidy simultaneously regulates the copy numbers, as well as the transcription levels, of the genes on the aneuploid chromosome. Specifically, in both FY1284 and FY1285, most *ERG* genes, including *ERG11*, had higher expression than was observed in the wild-type strain SC5314. Furthermore, both adaptors have higher expression of *CDR2* and *MDR1* (Table 1). It is noteworthy that some of these upregulated genes are on the aneuploidy chromosomes, whereas others are on the euploid chromosomes. Therefore, aneuploidy simultaneously regulates the expression of genes both on and outside the aneuploid chromosome. We posit that the aneuploid adaptors are preconditioned for exposure to FLC via the upregulation of the *ERG* genes and the drug efflux genes.

## DISCUSSION

In this study, we investigated how C. albicans adapted to the weak selection of FLC. We found the following characteristics of the adaptation procedure. First, short-time exposure to subinhibitory concentrations of FLC was sufficient to induce C. albicans to mount cellular responses, including the overexpression of *ERG* genes and drug efflux genes. Such an adaptive response was accompanied with aneuploidy formation in a subpopulation of cells. Second, aneuploidy happened at a fitness cost in the absence of FLC stress as well as in the presence of subinhibitory FLC, but it enabled the dose-independent tolerance of a subpopulation of cells to supra-MICs of FLC. Third, aneuploidy caused tolerance but not resistance to FLC. By DDA, the RAD_20_ values of the aneuploids were surprisingly larger than those of the parent, indicating that the aneuploids had not gained resistance to FLC. However, the FoG_20_ values of the aneuploids were much higher than those of the parent. The spot assays indicated that the aneuploids could grow at up to 128 μg/mL of FLC. Survival assays indicated that the tolerance was due to the FLC dose-independent growth of a subpopulation of cells. Fourth, the aneuploids were unstable. In the absence of FLC, the aneuploid adaptor with whole chromosome aneuploidy spontaneously reverted to whole chromosome euploidy, and the tolerance was concomitantly lost. Therefore, unlike genetic mutation, which is always nonreversible, aneuploidy-mediated drug tolerance can be reversible. In the adaptor with segmental aneuploidy, the segmental trisomy of ChrR was maintained, but the segmental monosomic region was unstable and was compensated by the duplication of the remaining homolog as well as by the duplication of another region on the same chromosome. The exact mechanism behind this complex aneuploidy is still under investigation. In summary, we found aneuploidy-mediated, rapid, and reversible adaptation of C. albicans to FLC.

In this study, we found that C. albicans adapted to FLC via unstable aneuploidy. In addition, in the model yeast Saccharomyces cerevisiae, some strains can form colonies that can switch between “fluffy” and “smooth” states. The parent strain was euploid and haploid. The disomy of Chr XVI was sufficient to cause the parent to switch from “fluffy” to “smooth” colonies, and the spontaneous loss of extra Chr XVI caused a reversion to “fluffy” colonies (22). Therefore, in addition to physiological and epigenetic mechanisms, at least in C. albicans and S. cerevisiae, the unstable genomic change of aneuploidy also causes reversible phenotype heterogeneity. C. albicans has a plastic genome. It tolerates the trisomy of each chromosome (23), but the aneuploidy state is unstable. In the absence of stress, aneuploids spontaneously and rapidly revert to euploidy (23, 24). Specific, strong stress usually causes particular aneuploidy formation in C. albicans (24–30) as well as in other fungi (31–33), and these topics are reviewed in reference (34).

In addition to strong selection, in this study, we found that short-time exposure to weak selection by subinhibitory FLC was sufficient to select for aneuploids. Furthermore, we previously found that other weak stresses were also sufficient to select for aneuploid adaptors in pathogenic fungi. In C. neoformans, we found that a sub-MIC of FLC selected mostly Chr1x2 adaptors (32). In C. albicans, we found that a sub-MIC of tunicamycin, an ER stress inducer, selected Chr2x3 adaptors (29). Therefore, we posit that aneuploidy-mediated adaptation in response to both weak and strong stresses is a widespread mechanism in fungi.

In yeast, in addition to the copy number, aneuploidy usually directly causes the proportional alteration of the transcript and protein levels of genes on the aneuploid chromosome (31, 35). Indirectly, aneuploidy can also alter the expression of genes on euploid chromosomes via the interference of the gene regulatory network. In this study, among the genes involved in ergosterol biosynthesis, *ERG13*, *ERG8*, *ERG26*, and *IDI1* are on the aneuploid chromosomes in one adaptor (FY1284), whereas *ERG25* and *ERG27* are on the aneuploid chromosome in both adaptors. In at least one of the adaptors, the expression of these *ERG* genes, as well as others, including *ERG1*, *ERG2*, *ERG3*, *ERG6*, *ERG7*, *ERG9*, ERG10, *ERG11*, *ERG12*, *ERG20*, and *ERG24* were upregulated. *CDR1*, *CDR2*, and *MDR1* encode drug efflux pumps. None of them were on the aneuploid chromosome. However, *CDR2* and *MDR1* were upregulated in both adaptors. Therefore, the aneuploidy adaptors directly and indirectly upregulated genes associated with ergosterol biosynthesis and drug efflux, thereby preparing themselves for further exposure to FLC.

In conclusion, we found that reversible genomic change via aneuploidy formation enabled rapid adaptation to a subinhibitory amount of FLC. The adaptation was accompanied with decreased fitness at low concentration of FLC, but it also conferred increased fitness at high concentrations of FLC via the constitutive upregulation of the genes associated with FLC tolerance. We posit that this novel strategy might be a general mechanism of adaption to stresses in human fungal pathogens.

## MATERIALS AND METHODS

### Strains and growth conditions.

Strains were stored in 20% glycerol at −80°C. Cells were grown either in YPD broth containing 1% [wt/vol] yeast extract, 2% [wt/vol] peptone, and 2% [wt/vol] d-glucose or on YPD-agar plates containing 2% [wt/vol] agar. Drugs were dissolved in dimethyl sulfoxide (DMSO) and stored at −20°C.

### Growth curve.

Approximately 2.5 × 10^3^ cells/mL of SC5314 in 150 μL YPD, with or without FLC, were incubated in a 96-well plate at 30°C. The OD_595_ value was monitored in a Tecan plate reader (Infinite F200 PRO, Tecan, Switzerland) at 15 min time intervals for 24 h. The data are presented as the mean ± SD of three biological replicates.

### Spot assay screening.

Approximately 2.5 × 10^3^ cells/mL of SC5314 were inoculated into 1.5 mL of YPD broth containing 0.5 μg/mL FLC. After 24 h of incubation with shaking, the culture was washed and diluted with distilled water. Approximately 300 cells were spread on YPD plates and were incubated at 30°C for 36 h. 120 colonies were randomly tested for tolerance to FLC.

### Spot assay.

Strains were streaked onto YPD-agar plates and incubated for 36 h. Several colonies were randomly chosen and suspended in distilled water. Cell densities were determined using a hemocytometer and were adjusted to 1 × 10^7^ cells/mL. Serial 10-fold dilutions of cell suspension were spotted (3 μL/spot) on plates supplemented with the drugs. The plates were incubated for 48 h and were then photographed.

### Disk diffusion assay.

The CLSI M44-A2 guidelines (36) for antifungal disk diffusion susceptibility testing were followed, with slight modifications. The strains were grown on YPD-agar plates. The cell density was adjusted to 1 × 10^6^ cells/mL using a hemocytometer. 100 μL of cell suspension were spread on plates. One empty paper disk was placed in the center of each plate. 5 μL of 5 mg/mL FLC solution were put on the disks. The plates were then incubated for 24 h and 48 h and photographed. The photographs were analyzed using the *diskImageR* pipeline (18). The means and standard deviations of the RAD_20_ and FoG_20_ values for three biological repeats were presented as point plots using a custom Python script.

### Survival assay.

Approximately 200 cells of each strain were spread on YPD-agar plates supplemented with FLC. The plates were incubated at 30°C for 48 h. The survival rate was calculated as the percentage of colonies on the drug plate versus on the YPD plate. The data are presented as the mean ± SD of three technical replicates.

### Colony instability.

FY1284 and FY1285 were streaked from −80°C to YPD-agar plates, and they were incubated at 30°C for 36 h. One small colony was randomly chosen and suspended in distilled water. The cells were diluted with distilled water. Approximately 200 cells were spread on a YPD-agar plate and were incubated at 30°C for 36 h. One small colony and one large colony were randomly chosen for further studies.

### Next-generation sequencing.

The DNA extraction, library construction, and sequencing were performed as previously described (23). The data were visualized using Ymap (37).

### RNA-seq.

**(i) Exposure to a subinhibitory concentration of FLC.** SC5314 was inoculated to a starting OD_600_ value of 0.2 in 50 mL YPD broth. The culture was incubated in a shaker at 30°C until the OD_600_ value reached 1.0. The culture was divided into two batches: control (only DMSO was added) and subinhibitory treatment (0.5 μg/mL FLC). After incubation with shaking for 3 h, the cultures were collected via centrifugation, washed, and flash frozen in liquid nitrogen.

**(ii) Comparison of adaptors and SC5314.** Strains were streaked onto YPD-agar plates. After incubation at 30°C for 36 h, several colonies were randomly chosen and suspended in distilled water. Cell densities were determined using a hemocytometer. Approximately 300 cells were spread onto YPD-agar plates. After incubation at 30°C for 36 h, the cells were collected and flash frozen in liquid nitrogen.

Three biological replicates were obtained for each condition. Total RNA extraction and purification, library construction, and sequencing were performed as previously described (29). The raw sequence files (.fastq files) underwent a quality control analysis using the FastQC tool (http://www.bioinformatics.babraham.ac.uk/projects/fastqc). The reads were mapped to the C. albicans strain SC5314 reference genome (Assembly 22) (http://www.candidagenome.org/download/sequence/C_albicans_SC5314/Assembly22/current/). The differential gene expression profiling was carried out using DESeq2 (38) with the standard parameters. Genes with FDR (false discovery rate)-adjusted *P* values of <0.05 and expression fold changes of more than 1.3 or less than −1.3 were considered to be differentially expressed.

### Statistical analysis.

The significance analysis of the differences between growth curves was performed using a paired, two-tailed *t* test using GraphPad Prism (version 5.01).

### Data availability.

The sequencing data have been deposited in the ArrayExpress database at EMBL-EBI (www.ebi.ac.uk/arrayexpress) under the accession numbers E-MTAB-11923, E-MTAB-11881, and E-MTAB-11890.

## References

[1] Horn DL, Neofytos D, Anaissie EJ, Fishman JA, Steinbach WJ, Olyaei AJ, Marr KA, Pfaller MA, Chang CH, Webster KM. 2009. Epidemiology and outcomes of candidemia in 2019 patients: data from the prospective antifungal therapy alliance registry. Clin Infect Dis 48:1695–1703. doi:10.1086/599039. 19441981

[2] Ruhnke M. 2006. Epidemiology of Candida albicans infections and role of non-Candida-albicans yeasts. Curr Drug Targets 7:495–504. doi:10.2174/138945006776359421. 16611037

[3] Pfaller MA, Diekema DJ. 2007. Epidemiology of invasive candidiasis: a persistent public health problem. Clin Microbiol Rev 20:133–163. doi:10.1128/CMR.00029-06. 17223626 PMC1797637

[4] Vanden Bossche H, Koymans L, Moereels H. 1995. P450 inhibitors of use in medical treatment: focus on mechanisms of action. Pharmacol Ther 67:79–100. doi:10.1016/0163-7258(95)00011-5. 7494862

[5] Bolard J. 1986. How do the polyene macrolide antibiotics affect the cellular membrane properties? Biochim Biophys Acta 864:257–304. doi:10.1016/0304-4157(86)90002-x. 3539192

[6] Kelly SL, Lamb DC, Corran AJ, Baldwin BC, Kelly DE. 1995. Mode of action and resistance to azole antifungals associated with the formation of 14 alpha-methylergosta-8,24(28)-dien-3 beta,6 alpha-diol. Biochem Biophys Res Commun 207:910–915. doi:10.1006/bbrc.1995.1272. 7864896

[7] Henry KW, Nickels JT, Edlind TD. 2000. Upregulation of ERG genes in Candida species by azoles and other sterol biosynthesis inhibitors. Antimicrob Agents Chemother 44:2693–2700. doi:10.1128/AAC.44.10.2693-2700.2000. 10991846 PMC90137

[8] Robbins N, Caplan T, Cowen LE. 2017. Molecular evolution of antifungal drug resistance. Annu Rev Microbiol 71:753–775. doi:10.1146/annurev-micro-030117-020345. 28886681

[9] Siikala E, Rautemaa R, Richardson M, Saxen H, Bowyer P, Sanglard D. 2010. Persistent Candida albicans colonization and molecular mechanisms of azole resistance in autoimmune polyendocrinopathy-candidiasis-ectodermal dystrophy (APECED) patients. J Antimicrob Chemother 65:2505–2513. doi:10.1093/jac/dkq354. 20876623

[10] Wirsching S, Michel S, Morschhäuser J. 2000. Targeted gene disruption in Candida albicans wild-type strains: the role of the MDR1 gene in fluconazole resistance of clinical Candida albicans isolates. Mol Microbiol 36:856–865. doi:10.1046/j.1365-2958.2000.01899.x. 10844673

[11] Anderson JB. 2005. Evolution of antifungal-drug resistance: mechanisms and pathogen fitness. Nat Rev Microbiol 3:547–556. doi:10.1038/nrmicro1179. 15953931

[12] Gullberg E, Cao S, Berg OG, Ilbäck C, Sandegren L, Hughes D, Andersson DI. 2011. Selection of resistant bacteria at very low antibiotic concentrations. PLoS Pathog 7:e1002158. doi:10.1371/journal.ppat.1002158. 21811410 PMC3141051

[13] Ramsay KA, McTavish SM, Wardell SJT, Lamont IL. 2021. The effects of sub-inhibitory antibiotic concentrations on Pseudomonas aeruginosa: reduced susceptibility due to mutations. Front Microbiol 12:789550. doi:10.3389/fmicb.2021.789550. 34987489 PMC8721600

[14] Nolan C, Behrends V. 2021. Sub-inhibitory antibiotic exposure and virulence in Pseudomonas aeruginosa. Antibiotics 10. doi:10.3390/antibiotics10111393. PMC8615142 34827331

[15] Rosenberg A, Ene IV, Bibi M, Zakin S, Segal ES, Ziv N, Dahan AM, Colombo AL, Bennett RJ, Berman J. 2018. Antifungal tolerance is a subpopulation effect distinct from resistance and is associated with persistent candidemia. Nat Commun 9:2470. doi:10.1038/s41467-018-04926-x. 29941885 PMC6018213

[16] Berman J, Krysan DJ. 2020. Drug resistance and tolerance in fungi. Nat Rev Microbiol 18:319–331. doi:10.1038/s41579-019-0322-2. 32047294 PMC7231573

[17] Xu Y, Lu H, Zhu S, Li WQ, Jiang YY, Berman J, Yang F. 2021. Multifactorial Mechanisms of tolerance to ketoconazole in Candida albicans. Microbiol Spectr 9:e0032121. doi:10.1128/Spectrum.00321-21. 34160280 PMC8552639

[18] Gerstein AC, Rosenberg A, Hecht I, Berman J. 2016. diskImageR: quantification of resistance and tolerance to antimicrobial drugs using disk diffusion assays. Microbiology (Reading) 162:1059–1068. doi:10.1099/mic.0.000295. 27126388 PMC5756480

[19] Yang F, Scopel EF, Li H, Sun L-l, Kawar N, Cao Y-b, Jiang Y-Y, Berman J. 2022. Antifungal tolerance and resistance emerge at distinct drug concentrations and rely upon different aneuploid chromosomes. bioRxiv. doi:10.1101/2022.11.30.518455. PMC10127634 36877011

[20] Selmecki A, Forche A, Berman J. 2006. Aneuploidy and isochromosome formation in drug-resistant Candida albicans. Science 313:367–370. doi:10.1126/science.1128242. 16857942 PMC1717021

[21] Ford CB, Funt JM, Abbey D, Issi L, Guiducci C, Martinez DA, Delorey T, Li BY, White TC, Cuomo C, Rao RP, Berman J, Thompson DA, Regev A. 2015. The evolution of drug resistance in clinical isolates of Candida albicans. Elife 4:e00662. doi:10.7554/eLife.00662. 25646566 PMC4383195

[22] Tan Z, Hays M, Cromie GA, Jeffery EW, Scott AC, Ahyong V, Sirr A, Skupin A, Dudley AM. 2013. Aneuploidy underlies a multicellular phenotypic switch. Proc Natl Acad Sci USA 110:12367–12372. doi:10.1073/pnas.1301047110. 23812752 PMC3725063

[23] Yang F, Todd RT, Selmecki A, Jiang YY, Cao YB, Berman J. 2021. The fitness costs and benefits of trisomy of each Candida albicans chromosome. Genetics 218. doi:10.1093/genetics/iyab056. PMC8225349 33837402

[24] Yang F, Teoh F, Tan ASM, Cao Y, Pavelka N, Berman J. 2019. Aneuploidy Enables cross-adaptation to unrelated drugs. Mol Biol Evol 36:1768–1782. doi:10.1093/molbev/msz104. 31028698 PMC6657732

[25] Janbon G, Sherman F, Rustchenko E. 1998. Monosomy of a specific chromosome determines L-sorbose utilization: a novel regulatory mechanism in Candida albicans. Proc Natl Acad Sci USA 95:5150–5155. doi:10.1073/pnas.95.9.5150. 9560244 PMC20229

[26] Selmecki A, Gerami-Nejad M, Paulson C, Forche A, Berman J. 2008. An isochromosome confers drug resistance in vivo by amplification of two genes, ERG11 and TAC1. Mol Microbiol 68:624–641. doi:10.1111/j.1365-2958.2008.06176.x. 18363649

[27] Yang F, Kravets A, Bethlendy G, Welle S, Rustchenko E. 2013. Chromosome 5 monosomy of Candida albicans controls susceptibility to various toxic agents, including major antifungals. Antimicrob Agents Chemother 57:5026–5036. doi:10.1128/AAC.00516-13. 23896475 PMC3811469

[28] Yang F, Zhang L, Wakabayashi H, Myers J, Jiang Y, Cao Y, Jimenez-Ortigosa C, Perlin DS, Rustchenko E. 2017. Tolerance to caspofungin in Candida albicans is associated with at least three distinctive mechanisms that govern expression of FKS genes and cell wall remodeling. Antimicrob Agents Chemother 61. doi:10.1128/AAC.00071-17. PMC5404545 28223384

[29] Yang F, Gritsenko V, Slor Futterman Y, Gao L, Zhen C, Lu H, Jiang YY, Berman J. 2021. Tunicamycin potentiates antifungal drug tolerance via aneuploidy in Candida albicans. mBio 12:e0227221. doi:10.1128/mBio.02272-21. 34465026 PMC8406271

[30] Ma Q, Ola M, Iracane E, Butler G. 2019. Susceptibility to medium-chain fatty acids is associated with trisomy of chromosome 7 in Candida albicans. mSphere 4. doi:10.1128/mSphere.00564-19. PMC6595153 31243082

[31] Sionov E, Lee H, Chang YC, Kwon-Chung KJ. 2010. Cryptococcus neoformans overcomes stress of azole drugs by formation of disomy in specific multiple chromosomes. PLoS Pathog 6:e1000848. doi:10.1371/journal.ppat.1000848. 20368972 PMC2848560

[32] Yang F, Gritsenko V, Lu H, Zhen C, Gao L, Berman J, Jiang YY. 2021. Adaptation to fluconazole via aneuploidy enables cross-adaptation to amphotericin B and flucytosine in Cryptococcus neoformans. Microbiol Spectr 9:e0072321. doi:10.1128/Spectrum.00723-21. 34585947 PMC8557924

[33] Yang F, Lu H, Wu H, Fang T, Berman J, Jiang YY. 2021. Aneuploidy underlies tolerance and cross-tolerance to drugs in Candida parapsilosis. Microbiol Spectr 9:e0050821. doi:10.1128/Spectrum.00508-21. 34612700 PMC8510177

[34] Tsai HJ, Nelliat A. 2019. A double-edged sword: aneuploidy is a prevalent strategy in fungal adaptation. Genes 10. doi:10.3390/genes10100787. PMC6826469 31658789

[35] Pavelka N, Rancati G, Zhu J, Bradford WD, Saraf A, Florens L, Sanderson BW, Hattem GL, Li R. 2010. Aneuploidy confers quantitative proteome changes and phenotypic variation in budding yeast. Nature 468:321–325. doi:10.1038/nature09529. 20962780 PMC2978756

[36] CLSI. 2009. Method for antifungal disk diffusion susceptibility testing of yeasts, 2nd ed Clinical and Laboratory Standards Institute, Wayne, PA.

[37] Abbey DA, Funt J, Lurie-Weinberger MN, Thompson DA, Regev A, Myers CL, Berman J. 2014. YMAP: a pipeline for visualization of copy number variation and loss of heterozygosity in eukaryotic pathogens. Genome Med 6:100. doi:10.1186/PREACCEPT-1207699561372700. 25505934 PMC4263066

[38] Love MI, Huber W, Anders S. 2014. Moderated estimation of fold change and dispersion for RNA-seq data with DESeq2. Genome Biol 15:550. doi:10.1186/s13059-014-0550-8. 25516281 PMC4302049

