# Supplementary material for: Aneuploidy Mediates Rapid Adaptation to a Subinhibitory Amount of Fluconazole in Candida albicans
Source: Microbiol Spectr. 2023 Feb 28;11(2):e03016-22. doi: 10.1128/spectrum.03016-22 (PMC10101127; doi:10.1128/spectrum.03016-22)
Supplement: Supplemental file 3 — Fig. S1. Download spectrum.03016-22-s0003.pdf, PDF file, 7.0 MB [file spectrum.03016-22-s0003.pdf]

Supplemental materials

Figure S1. Short time exposure to sub-inhibitory concentration of fluconazole selects tolerant adaptors.

C. albicans lab strain SC5314 was grown in YPD broth with or without 0.5 μg/ml fluconazole (FLC) for 24h. Randomly 120 colonies isolated from the cultures were tested for tolerance to FLC by spot assay. Magenta circles indicate the parent strain. Cyan circles indicate the two tolerant adaptors. The plates were incubated at 30˚C for 48 h then photographed.

Table S1. List of differentially expressed genes induced by exposure of SC5314 to sub-inhibitory amount of fluconazole.

Table S2. List of GO term enrichment analysis of the differential genes.

Figure S1

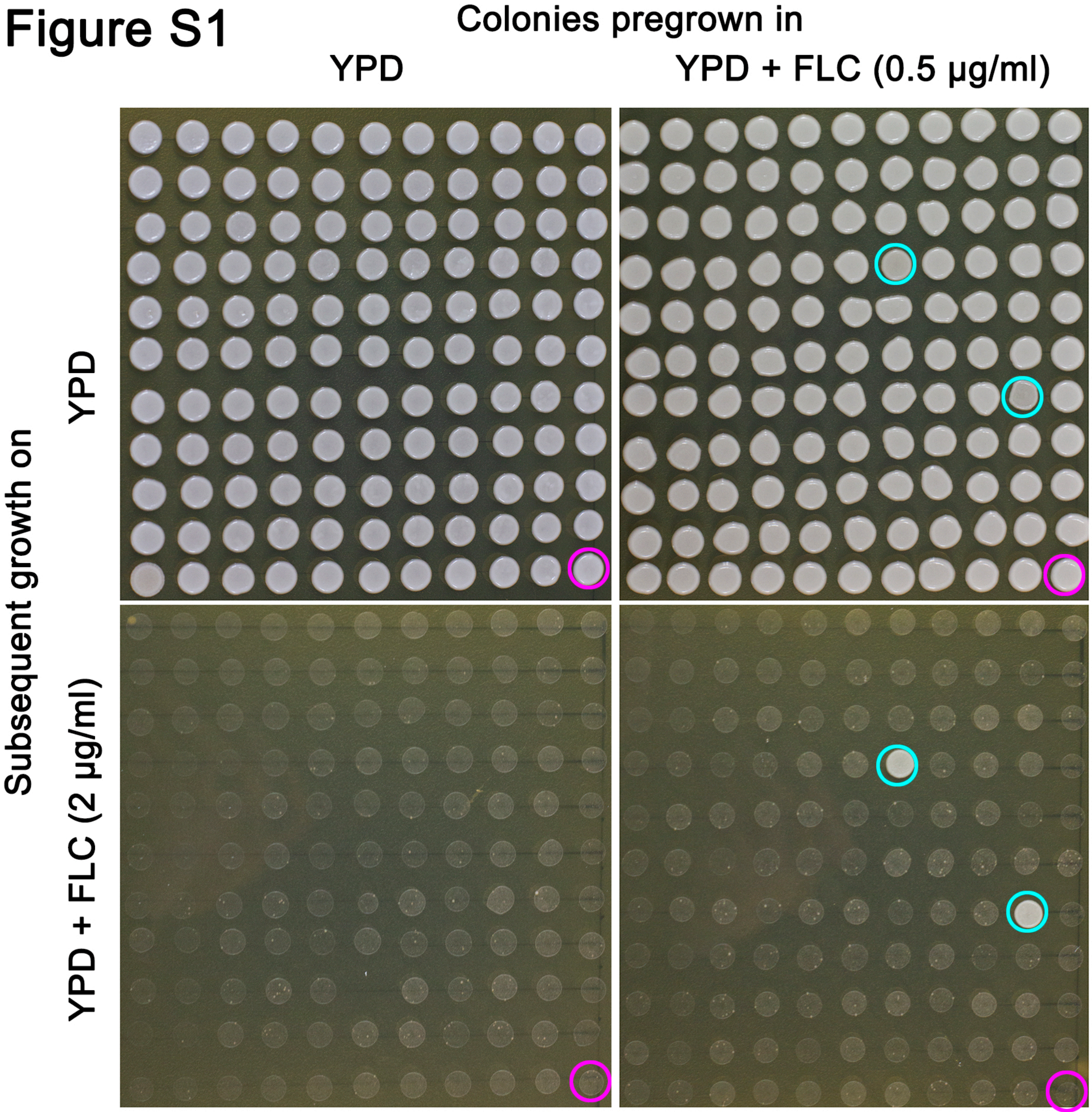

Colonies pregrown in
YPD
YPD + FLC (0.5 µg/ml)
Subsequent growth on
YPD
YPD + FLC (2 µg/ml)
